# Validated determination method of tramadol and its desmethylates in human plasma using an isocratic LC-MS/MS and its clinical application to patients with cancer pain or non-cancer pain

**DOI:** 10.1186/s40780-016-0059-2

**Published:** 2016-10-04

**Authors:** Hironari Tanaka, Takafumi Naito, Yasuaki Mino, Junichi Kawakami

**Affiliations:** Department of Hospital Pharmacy, Hamamatsu University School of Medicine, 1-20-1 Handayama, Higashi-ku, Hamamatsu, Shizuoka 431-3192 Japan

**Keywords:** Tramadol, Desmethylate, LC-MS/MS, Human plasma, Pharmacokinetics

## Abstract

**Background:**

This study aimed to develop a simultaneous determination method for tramadol and its desmethylates in human plasma using isocratic liquid chromatography coupled to tandem mass spectrometry and to validate it for pharmacokinetic evaluation in patients with cancer pain or non-cancer pain.

**Methods:**

The pretreatments for human plasma involved protein precipitation using acetonitrile and methanol under basic conditions. Tramadol, *O*-desmethylate, *N*-desmethylate, and *N,O*-didesmethylate were separated on an octadecylsilyl column filled with 3-μm particles using isocratic mixture of methanol and 0.15 % formic acid in water (35:65, v/v). The mass spectrometer was run in positive ion multiple reaction monitoring mode. This method was applied to the determination of plasma samples in patients treated with oral tramadol.

**Results:**

The chromatographic total run time was 10 min. The calibration curves in human plasma of tramadol, *O*-desmethylate, *N*-desmethylate, and *N,O*-didesmethylate were linear over the concentration ranges of 12.5–1600, 2.5–320, 2.5–320, and 2.5–320 ng/mL, respectively. The lower limits of quantitation of tramadol and its desmethylates in human plasma were 12.5 and 2.5 ng/mL. Their extraction recoveries were 85.5–106.3 %. The intra-day and inter-day precisions and accuracies were 1.6–10.2 % and 89.2–106.2 % for all analytes. The plasma concentration ranges of tramadol, *O*-desmethylate, *N*-desmethylate, and *N,O*-didesmethylate were 18.2–564, 11.8–137, 4.9–250, and 6.1–147 ng/mL in cancer patients, and 32.8–670, 7.0–84.8, 5.1–317, and 6.7–85.2 ng/mL, respectively, in non-cancer patients.

**Conclusions:**

The present method with acceptable analytical performance can be helpful for evaluating the pharmacokinetics of oral tramadol, including the determination of its desmethylates, for patients with cancer pain or non-cancer pain in clinical settings.

## Background

Tramadol, a centrally acting analgesic agent, is commonly used for the treatment of cancer pain and non-cancer pain as an alternative to opioid analgesics [1]. Tramadol dually acts as an opioid μ1 receptor agonist and a monoamine reuptake inhibitor [2, 3]. Based on these actions, tramadol is effective for complicated pain associated with neuropathic disorders. The common adverse effects of tramadol are somnolence, nausea, and vomiting. Serious adverse effects involving seizures and serotonin syndrome potentially also occur with therapeutic doses of tramadol [4]. The incidence of these adverse effects can lead to drug withdrawal or poor pain control. The analgesic and adverse effects of tramadol show a large interindividual variability in patients with cancer pain or non-cancer pain [5].

Tramadol is rapidly absorbed from the intestine after oral administration and its oral bioavailability is 65–70 % due to first-pass metabolism [6]. Tramadol is metabolized to *O*-desmethyltramadol (ODT) primarily by cytochrome P450 (CYP) 2D6, and *N*-desmethyltramadol (NDT) by CYP2B6 and CYP3A4. ODT and NDT are further metabolized to *N,O*-didesmethyltramadol (NODT) by CYPs [7]. ODT has a 700-fold higher affinity towards opioid μ1 receptors than tramadol and is the main contributor to the analgesic efficacy of tramadol pharmaceuticals. NDT and NODT have a weak affinity for opioid μ1 receptors and weak inhibition of monoamine reuptake [8]. The pharmacokinetics of tramadol and its desmethylates show a large interindividual variability in humans [9]. In addition, the relationships between the plasma concentrations of tramadol and its desmethylates and clinical effects remain to be clarified in clinical settings.

Tramadol and its desmethylates in human plasma have been determined using several chromatographic techniques such as liquid chromatography (LC) coupled to ultraviolet or fluorescence detection, and LC coupled to tandem mass spectrometry (MS/MS) [10–12]. In general, ultraviolet detection from biological specimens such as plasma and urine is not suitable because of low sensitivity and selectivity [13, 14]. The LC separation of tramadol, ODT, NDT, and NODT using ultraviolet or fluorescence detection requires surfactants such as triethylamine and sodium dodecyl sulfate [15, 16]. These surfactants cause the ionic suppression of analytes in MS/MS analysis. MS/MS detection of tramadol and its desmethylates possesses high sensitivity and selectivity. However, distinguishing between ODT and NDT in MS/MS analysis requires LC separation due to similar molecular mass and fragmentation patterns. LC-MS/MS has a limit with regards to the selection of the mobile phase because of poor ionization of the desmethylates. To date, few practical methods using simultaneous LC-MS/MS are available for the determination of tramadol and its desmethylates in human plasma.

The potential pharmacokinetic differences between cancer and non-cancer patients were observed in recent reports [17, 18]. However, few validated method is available for the determination of tramadol and its desmethylates in human plasma in patients with non-cancer pain. The development of effective and validated chromatographic methodologies for the determination of tramadol and its desmethylates in human specimens is needed for clinical use. This study aimed to develop a simultaneous determination method for tramadol and its desmethylates in human plasma using an isocratic LC-MS/MS. The method was validated in terms of pharmacokinetic evaluation in patients with cancer pain and patients with non-cancer pain.

## Methods

### Materials

Tramadol, ODT, NDT, NODT, and tramadol*-d6* as an internal standard (IS) were obtained from Toronto Research Chemicals Inc. (Toronto, Ontario, Canada). HPLC-grade methanol and 28 % ammonia solution were purchased from Wako Pure Chemicals (Osaka, Japan). All other reagents were of analytical grade and commercially available.

### Solutions

Stock solutions of tramadol (100 μg/mL), ODT (50 μg/mL), NDT (50 μg/mL), NODT (20 μg/mL), and IS (20 μg/mL) were prepared in methanol. Standard solutions of tramadol, ODT, NDT, and NODT were obtained by the dilution of stock solution with methanol. Calibration standards were prepared in drug-free pooled plasma (Kohjin-Bio Co., Ltd, Sakado, Japan). The final concentrations of tramadol were 12.5, 25, 50, 100, 200, 400, 800, and 1600 ng/mL, while those of ODT, NDT, and NODT were 2.5, 5, 10, 20, 40, 80, 160, and 320 ng/mL. Quality control (QC) samples were spiked to tramadol concentrations of 12.5, 50, 200, and 800 ng/mL and ODT, NDT, and NODT concentrations of 2.5, 10, 40, and 160 ng/mL in drug-free plasma.

### Sample pretreatment

Blood specimens were collected into EDTA dipotassium salt (2 K) tubes. Plasma was obtained by centrifugation of the blood at 1670 × *g* at 4 °C for 10 min and then stored at −80 °C until sample pretreatment. To 100 μL aliquots of plasma, 600 μL of acetonitrile, 100 μL of IS solution (50 ng/mL), and 20 μL of 28 % ammonia solution were added into a microtube. After 30 min on a vortex mixer, the mixtures were stored at −35 °C for 30 min and ultrasonicated for 30 min. The mixtures were centrifuged at 17,900 × *g* at 4 °C for 20 min, and then 750 μL of the supernatant was evaporated to dryness by rotary vacuum evaporation without heating. The residues were reconstituted with 150 μL of mixture containing methanol and 0.15 % formic acid in water (1:1, v/v). After 30 min on a vortex mixer, the mixtures were ultrasonicated for 30 min. The mixtures were centrifuged at 17,900 × *g* at 4 °C for 20 min. The supernatants were filtrated with a Millex-LH syringe filter (0.45 μm, 4 mm, Merck Millipore Ltd., Billerica, MA, USA) before injection into the LC.

### Chromatographic conditions

Tramadol, ODT, NDT, NODT, and IS in human plasma were separated using a validated LC system (UFLC_XR_, Shimadzu Corporation, Kyoto, Japan). The LC system consisted of a CBM-20A system controller, DGU-20A_5R_ degasser, LC-20AD_XR_ pump, SIL-20AC_XR_ autoinjector, and CTO-20AC column oven. Separation was performed using a 3-μm particle ODS column (TSKgel ODS-100 V, 150 × 2.0 mm I.D., Tosoh, Tokyo, Japan) with a guard column (TSKguardgel ODS-100 V, 3 μm particle size, 10 × 2.0 mm I.D., Tosoh). The mobile phase consisted of methanol and 0.15 % formic acid in water (35:65, v/v). The flow rate was 0.2 mL/min and the column temperature was set at 40 °C, and the autoinjector was set at 4 °C. The injection volume was 10 μL.

### Mass spectrometric conditions

**Fig. 1:**
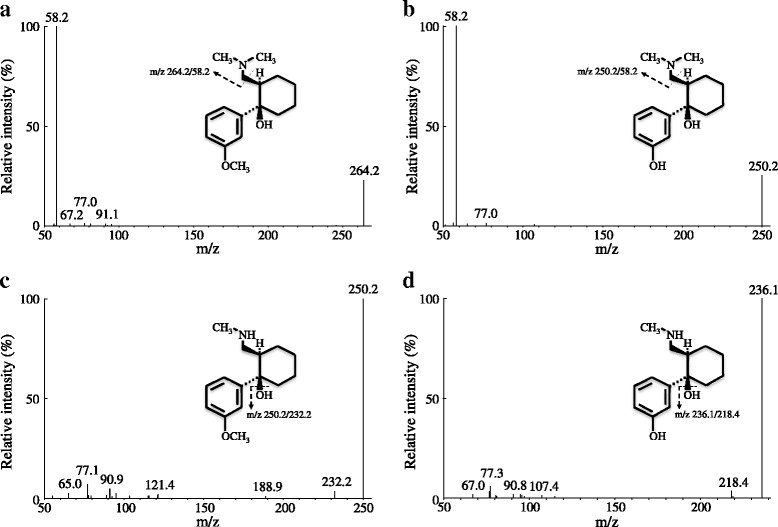
Mass spectra of tramadol (**a**), *O*-desmethylate (**b**), *N*-desmethylate (**c**), and *N,O*-didesmethylate (**d**). A mass-to-charge (*m/z*) of 264.2/58.2 was monitored for tramadol, 250.2/58.2 for *O*-desmethylate, 250.2/232.2 for *N*-desmethylate, and 236.1/218.4 for *N,O*-didesmethylate

The column effluent was monitored using a triple quadrupole mass spectrometer (3200 QTRAP®, AB Sciex, Foster City, CA, USA) equipped with an electrospray probe in positive ionization mode. It was controlled by Analyst software Version 1.6.1 (AB Sciex). The ion transitions were monitored using a dwell time of 200 milliseconds for each compound: tramadol, 264.2/58.2; ODT, 250.2/58.2; NDT, 250.2/232.2; NODT, 236.1/218.4; and IS, 270.2/64.1 (Fig. 1). Samples were introduced to the interface through a turbo ion spray with the temperature set at 600 °C. A high positive voltage of 5.5 kV was applied to the ion spray. Collision gas, curtain gas, ion source gas 1, and ion source gas 2 were set at 5 psi, 30 psi, 60 psi, and 60 psi, respectively. Collision energy for tramadol, ODT, NDT, NODT, and tramadol*-d6* were −31, −31, −13, −13, and −35 V, respectively.

### Method validation

Selectivity of the method was evaluated by analyzing six independent drug free plasma samples. Calibration curves were obtained by plotting the measured peak area ratios of tramadol, ODT, NDT, and NODT to IS. The linearities of tramadol, ODT, NDT, and NODT were observed at concentration ranges of 12.5–1600, 2.5–320, 2.5–320, and 2.5–320 ng/mL, respectively. Accuracy and precision were calculated for four QC samples in plasma. The lower limit of quantification (LLOQ) was defined as the concentration at which the relative standard deviation (RSD) does not exceed 20 %. Accuracies were determined by evaluating the analytical recovery of known amounts of plasma specimens. The intra-assay and inter-assay precisions were expressed as the RSD. Pretreatment recovery and matrix effect were assessed by three and five replicates of spiked human plasma at 25–400 and 5–80 ng/mL of tramadol and its desmethylates, respectively. The stabilities of analytes in plasma were evaluated by comparing peak areas after 24 h of storage at 4 °C and room temperature with initial peak area. Long-term stabilities in plasma at −80 °C were determined after 1 month. Analytical stabilities in injection solutions were evaluated by comparing peak areas after 24 h of storage at 4 °C with initial peak area.

### Patients and pharmacokinetic evaluation

A total of 30 Japanese patients, 15 with cancer pain and 15 with non-cancer pain, treated with oral tramadol at Hamamatsu University Hospital were enrolled (Table 1). The patients received tramadol oral dispersing tablets (Tramal®, Nippon Shinyaku Co., Ltd., Kyoto) or tramadol combination tablets (Tramcet combination Tablets®, Janssen Pharmaceutical K.K., Tokyo) four times a day for cancer pain and three times a day for non-cancer pain. The median daily dose was 100 mg for cancer pain and 112.5 mg for non-cancer pain. No patient was co-treated with potent enzyme modifiers such as an azole antifungal agent or rifampicin. Two mL blood samples were collected at 8 h post-dose (before breakfast) on the 4th day after initiation of therapy or later. The plasma concentrations of tramadol and its desmethylates were evaluated as the trough plasma concentration and the trough adjusted values. The metabolism of tramadol was estimated using the ratio of the plasma concentration of the desmethylates to tramadol as the metabolic ratio.

## Results

### Separation and selectivity

**Fig. 2:**
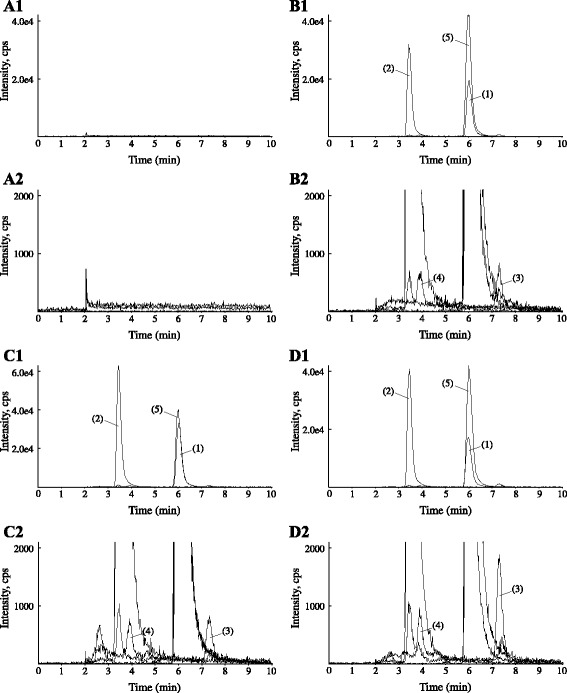
MS/MS chromatograms of human drug-free plasma (**a**), human drug-free plasma spiked with 200 ng/mL tramadol, 40 ng/mL *O*-desmethylate, 40 ng/mL *N*-desmethylate, and 40 ng/mL *N,O*-didesmethylate, (**b**) a plasma specimen at 8 h after evening dosing in cancer pain (**c**) and non-cancer pain (**d**) patients treated with oral tramadol. (1) Tramadol, (2) *O*-desmethylate, (3) *N*-desmethylate, (4) *N,O*-didesmethylate, and (5) tramadol-*d6* as internal standard

Figure 2 shows the LC-MS/MS chromatograms of tramadol, ODT, NDT, NODT, and IS in human plasma. No peaks interfering with tramadol, ODT, NDT, NODT, or IS in six independent drug-free plasma specimens in cancer and non-cancer patients were observed (Fig. 2a). Tramadol, ODT, NDT, NODT, and IS were eluted at 6.1, 3.4, 7.4, 3.9, and 6.0 min, respectively, with a total run time of 10 min (Fig. 2b). In addition, no peaks interfering with detection in tramadol non-treated patients with cancer pain or non-cancer pain were observed (Fig. 2c and d).

### Calibration curve, sensitivity, recovery, and matrix effect

The calibration curves of tramadol, ODT, NDT, and NODT in human plasma were linear over the concentration ranges of 12.5–1600, 2.5–320, 2.5–320, and 2.5–320 ng/mL, respectively. Their correlation coefficients were greater than 0.999. The LLOQ of tramadol, ODT, NDT, and NODT in human plasma were 12.5, 2.5, 2.5, and 2.5 ng/mL, respectively (n = 6). The pretreatment recoveries including deproteinization of tramadol, ODT, NDT, and NODT were mean ± standard deviation (SD), 86.0 ± 3.4 %, 85.5 ± 1.8 %, 106.3 ± 2.9 %, and 93.9 ± 0.8 %, respectively. The analytes and IS did not exhibit any matrix effects in human plasma (mean ± SD, 88.3 ± 4.1 % for tramadol, 89.9 ± 5.6 % for ODT, 105.1 ± 2.7 % for NDT, 98.8 ± 6.4 % for NODT, and 91.7 ± 4.9 % for IS, n = 5).

### Assay accuracy and precision in human plasma

Table 2 shows the intra- and inter-assay accuracies and precisions in human plasma. The intra-assay and inter-assay accuracies of tramadol, ODT, NDT, and NODT were 102.0–106.2 % and 95.1–103.4 %, 93.4–102.0 % and 94.9–100.8 %, 89.2–105.2 % and 92.7–101.6 %, and 92.5–102.5 % and 97.5–99.8 %, respectively. The intra-assay and inter-assay precisions of tramadol, ODT, NDT, and NODT were 1.6–8.2 % and 4.6–6.3 %, 3.6–4.8 % and 2.7–5.1 %, 3.4–7.9 % and 3.2–6.3 %, and 6.2–8.7 % and 4.2–10.2 %, respectively.

**Table 1:** Patient characteristics

	Cancer	Non-cancer
Gender, male/female	12/3	8/7
Age (years)	69 (66–73)	68 (67–77)
Body weight (kg)	44.9 (41.8–56.4)	55.9 (48.9–62.4)
Total protein (g/dL)	6.3 (6.0–6.5)	6.9 (6.0–7.5)
Serum albumin (g/dL)	3.2 (3.0–3.7)	3.1 (3.0–3.8)
Serum creatinine (mg/dL)	0.81 (0.65–0.93)	0.79 (0.59–0.95)
Blood urea nitrogen (mg/dL)	16.0 (13.4–19.2)	15.4 (14.1–20.1)
Total bilirubin (mg/dL)	0.4 (0.3–0.5)	0.5 (0.4–0.7)
Aspartate aminotransferase (IU/L)	18 (16–28)	28 (22–46)
Alanine aminotransferase (IU/L)	19 (13–35)	25 (17–41)

Data are expressed as median and interquartile range in parentheses

**Table 2:** Intra- and inter-assay precisions and accuracies of tramadol and its desmethylates in human plasma

Sample analytes	Theoretical value (ng/mL)	Intra-assay (n = 6)			Inter-assay (n = 6)		
		Mean ± SD (ng/mL)	Accuracy (%)	RSD (%)	Mean ± SD (ng/mL)	Accuracy (%)	RSD (%)
Tramadol	12.5	13.3 ± 0.71	106.2	4.8	12.6 ± 0.87	101.2	6.3
	50	51.7 ± 4.69	103.3	8.2	51.8 ± 3.49	103.4	6.0
	200	203.7 ± 12.9	102.0	6.0	204.2 ± 10.5	102.2	4.8
*O*-desmethylate	800	819.8 ± 13.1	102.5	1.6	761.3 ± 39.3	95.1	4.6
	2.5	2.49 ± 0.14	99.5	4.8	2.52 ± 0.08	100.8	2.7
	10	9.67 ± 0.38	96.7	3.6	9.90 ± 0.39	99.0	3.6
	40	37.4 ± 1.82	93.4	4.5	38.0 ± 2.14	94.9	5.1
*N*-desmethylate	160	162.8 ± 7.52	102.0	4.4	159.2 ± 6.46	99.7	3.8
	2.5	2.23 ± 0.20	89.2	7.9	2.41 ± 0.09	96.5	3.2
	10	10.5 ± 0.40	105.2	3.4	10.2 ± 0.70	101.6	6.3
	40	37.3 ± 2.66	93.3	6.5	37.1 ± 1.60	92.7	4.0
*N,O*-desmethylate	160	157.0 ± 8.83	98.2	5.2	155.0 ± 8.12	96.8	4.8
	2.5	2.32 ± 0.22	92.7	8.7	2.49 ± 0.17	99.8	6.2
	10	9.2 ± 0.77	92.5	7.6	9.77 ± 1.09	97.7	10.2
	40	41.0 ± 3.09	102.2	6.9	39.5 ± 2.71	98.8	6.3
	160	164.2 ± 11.1	102.5	6.2	155.8 ± 6.79	97.5	4.2

*SD* standard deviation, and *RSD* relative standard deviation

### Stability tests

The stock solutions of tramadol, ODT, NDT, NODT, and IS were stable at 4 °C (% of initial value, 88.3–99.1 %) for up to 3 months. Tramadol, ODT, NDT, and NODT in plasma specimens were stable at room temperature (% of initial value, 88.1–113.3 %) for up to 24 h. Tramadol, ODT, NDT, and NODT in plasma specimens were stable at −80 °C (% of initial value, 89.9–111.8 %) for up to 1 month. Tramadol, ODT, NDT, NODT, and IS in injection solutions were stable at 4 °C (% of initial value, 92.6–104.6 %) for up to 24 h.

### Plasma concentrations of tramadol and its desmethylates

**Fig. 3:**
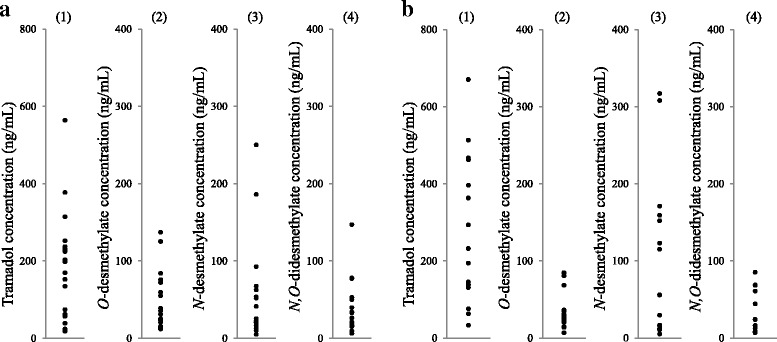
Plasma concentrations of tramadol, *O*-desmethylate, *N*-desmethylate, and *N,O*-didesmethylate obtained from patients with cancer pain (**a**) or non-cancer pain (**b**) just before treatment of oral tramadol on day 4 or later. (1) Tramadol, (2) *O*-desmethylate, (3) *N*-desmethylate, and (4) *N,O*-didesmethylate

Figure 3 shows the plasma concentrations of tramadol and its desmethylates in cancer pain and non-cancer pain patients treated with oral tramadol. The plasma concentrations of tramadol, ODT, NDT, and NODT in patients with cancer pain ranged from 18.2 to 564, 11.8 to 137, 4.9 to 250, and 6.1 to 147 ng/mL, respectively. In patients with non-cancer pain, the plasma concentrations of tramadol, ODT, NDT, and NODT ranged from 32.8 to 670, 7.0 to 84.8, 5.1 to 317, and 6.7 to 85.2 ng/mL, respectively. The plasma concentration ranges of tramadol and its desmethylates were measurable within their calibration curves in cancer and non-cancer patients.

### Variations in plasma exposure and metabolic ratio

The median and interquartile range (IQR) of dose-adjusted plasma concentrations for tramadol, ODT, NDT, and NODT were 73.6 and 33.4–88.2, 14.8 and 9.1–34.0, 14.0 and 7.9–26.1, and 12.3 and 7.5–18.8 ng/mL per mg/kg in patients with cancer pain, respectively. In patients with non-cancer pain, the median dose-adjusted plasma concentrations of tramadol, ODT, NDT, and NODT were 122 (IQR, 96.2–180), 19.2 (10.3–20.7), 29.1 (10.6–78.5), and 10.4 (5.2–20.7) ng/mL per mg/kg, respectively. The median metabolic ratios to ODT, NDT, and NODT were 0.30 (IQR, 0.22–0.36), 0.25 (0.15–0.43), and 0.23 (0.12–0.32) in patients with cancer pain, respectively. In patients with non-cancer pain, the median metabolic ratios to ODT, NDT, and NODT were 0.15 (IQR, 0.09–0.22), 0.19 (0.13–0.51), and 0.09 (0.06–0.19), respectively.

## Discussion

Development of effective and practical chromatographic methodologies for the determination of tramadol and its desmethylates in human specimens is needed for clinical use. This study developed a simultaneous determination method for tramadol and its desmethylates in human plasma using an isocratic LC-MS/MS and to evaluate its clinical suitability in patients with cancer pain and non-cancer pain. The chromatographic run time was 10 min. The calibration curves of tramadol and its desmethylates in human plasma were linear over the concentration ranges of 12.5–1600 and 2.5–320 ng/mL, respectively. The accuracy and precision data obtained with this method met the standards of an international guideline [19]. The plasma concentration ranges of tramadol and its desmethylates were measurable within their calibration curves in cancer and non-cancer patients. The present method with acceptable analytical performance can be helpful for evaluating the pharmacokinetics of tramadol in patients with cancer pain or non-cancer pain in clinical settings.

The pretreatments for human plasma involved protein precipitation using acetonitrile and methanol under basic conditions. The pretreatment recoveries of tramadol and its desmethylates in the present method were more than 85 %. Ardakani *et al.* reported on liquid-liquid extraction from plasma specimens using ethyl acetate under basic conditions [11]. The pretreatment recoveries of tramadol and ODT ranged from 74.7 to 80.8 % and 76.9 to 87.3 %, respectively. Liquid-liquid extraction using *tert*-butylmethyl ether and ethyl acetate with ammonium solution was also described [20]. In this pretreatment, the recoveries of tramadol and ODT ranged from 70 to 86 %. Sample pretreatment under basic conditions achieves high and stable pretreatment recoveries because the acid dissociation constant (pKa) of tramadol is 9.4. The present simple pretreatment without liquid-liquid extraction employed ammonium solution as a basic volatile reagent. The ammonium solution did not affect the MS/MS analysis owing to the evaporation of precipitate solution. These data indicate that the deproteinization including ammonia solution is suitable for the clean-up of tramadol and its desmethylates in human plasma.

The LLOQs of the present method for tramadol, ODT, NDT, and NODT were 12.5, 2.5, 2.5, and 2.5 ng/mL in human plasma, respectively. The LLOQ was defined as the concentration at which the RSD does not exceed 20 %. A validated method with sensitivity of 10 ng/mL for tramadol and 2.5 ng/mL for desmethylates is needed for evaluating the pharmacokinetics of oral tramadol in clinical settings. Ardakani *et al.* reported the simultaneous determination of tramadol, ODT, NDT, and NODT using HPLC-fluorescence detection [11]. The sensitivity of our present MS/MS method was similar to that of their HPLC-fluorescence method. Patel *et al.* described an LC-MS/MS method with LLOQs of 1 ng/mL for tramadol and 0.5 ng/mL for ODT [12]. Figure 2b shows the MS/MS chromatograms of human drug-free plasma spiked with 200 ng/mL tramadol, 40 ng/mL ODT, 40 ng/mL NDT, and 40 ng/mL NODT. Since NDT and NODT have less ionized property than ODT in mobile phase, our method is optimized for the MS/MS detection of NDT and NODT. Meyer *et al*. developed the LC-MS/MS method for the determination of plasma tramadol and ODT with LLOQ of 1 ng/mL [21]. This method has no analytical conditions for the determination of plasma NDT and NODT. The present LLOQs are sufficient to determine the plasma concentrations of tramadol and its desmethylates in clinical settings. Our method can determine the plasma tramadol and its desmethylates in patients treated with oral tramadol.

The run time for the LC separation was 10 min in this study. Our method determined tramadol and its desmethylates isocratically using a conventional ODS column with 3-μm particle size. The mobile phase consisted of methanol and 0.15 % formic acid without nonvolatile salts. Haage *et al*. determined the enantiomers of tramadol and its three main desmethylates in whole blood using an LC-MS/MS [22]. The chromatographic run time was approximately 30 min. Ardakani *et al.* employed a non-particle Chromolith® high-resolution column and the run time for LC separation was 7 min [11]. In their method, the mobile phase consisted of methanol and nonvolatile phosphoric acid salts solution. Since tramadol is a basic drug with a pKa of 9.4, a basic mobile phase is better than an acidic mobile phase in the LC separation. The other methods also used a mobile phase that included phosphate buffer, which is not suitable for MS/MS analysis [15, 20]. In contrast, the sensitivity in MS/MS detection for tramadol and its desmethylates declined under basic mobile phase. In MS/MS detection, two metabolites, ODT and NDT, had similar molecular masses and fragmentation patterns. ODT was detectable under the MS/MS condition of NDT. The retention times of ODT and NDT were 3.4 and 7.4 min, respectively, and the present method can adequately separate these two metabolites. The present method using an isocratic LC-MS/MS achieves the simple and rapid determination of tramadol and its desmethylates.

The precisions and accuracies of the present method in human plasma for tramadol and its desmethylates were within 10.9 % and 89.2–106.2 %, respectively. Tramadol and its desmethylates in plasma specimens could be stored at room temperature for up to 24 h and at −80 °C for up to 1 month. Tramadol and its desmethylates were stable under the pretreatment and measurement conditions. Many samples can be determined with the method because the analytes were stable in injection solutions for up to 24 h after preparation. More than 500 chromatographic runs were possible with one ODS column without any deterioration in separation performance. The results obtained with this method met the standards of the international US FDA guideline [19]. This validated method can be utilized to evaluate the pharmacokinetics of tramadol and its desmethylates in clinical settings.

The trough plasma concentration ranges of tramadol, ODT, NDT, and NODT were 18.2–564, 11.8–137, 4.9–250, and 6.1–147 ng/mL in cancer patients, and 32.8–670, 7.0–84.8, 5.1–317, and 6.7–85.2 ng/mL, respectively, in non-cancer patients. The calibration curves of tramadol, ODT, NDT, and NODT in human plasma were linear over the concentration ranges of 12.5–1600, 2.5–320, 2.5–320, and 2.5–320 ng/mL, respectively. The plasma concentration ranges of tramadol and ODT in patients receiving 200 mg of oral tramadol were 100–300 and 40–90 ng/mL, respectively [23]. The plasma concentration ranges of tramadol and ODT were measurable within the present calibration curves. The present method is able to determine the peak concentrations of tramadol and ODT.

The trough plasma concentrations of tramadol, ODT, NDT, and NODT in patients with cancer pain or non-cancer pain showed a large variability in this study. In addition, their dose-adjusted values and metabolic ratio to tramadol desmethylates also had a large individual variation in both populations. Tramadol is a substrate of CYP2D6, CYP2B6, and CYP3A4 and is rapidly and extensively metabolized in the liver [24]. In patients with cancer pain or non-cancer pain, the trough plasma concentrations of tramadol, ODT, NDT, and NODT were not shown for each genotype. Siew *et al.* demonstrated that genetic variants of CYP2D6 affected the pharmacokinetics and adverse effects of tramadol [10]. In future studies, the impact of CYP2D6 genetic variants on the plasma concentrations of tramadol and its desmethylates and clinical effects should be evaluated in patients with cancer pain or non-cancer pain. In addition, some patients treated with oral tramadol potentially have cancer cachexia in the present study population. Our previous reports demonstrated that cancer cachexia decreases the activity of cytochrome P450 [25, 26]. The difference in the dose-normalized plasma concentration of tramadol and its desmethylates between the patients receiving tramadol oral dispersing tablets and those receiving tramadol combination tablets were not observed in this study population (data not shown). Based on our data, the cancer cachexia may not strongly affect the plasma exposure of tramadol and its desmethylates in the enrolled patients.

The present study has several limitations. First, application of the present method is limited to patients receiving oral tramadol. Oral tramadol undergoes extensive first-pass metabolism in the liver. In patients treated with intravenous tramadol, the present method was not verified for suitability in this report. Second, the present method did not evaluate the suitability for special populations. Tramadol is eliminated by hepatic metabolism and renal excretion. The present method needs to be verified in patients with severe renal impairment or hepatic dysfunction. Third, this study did not characterize the difference in the plasma exposure of tramadol and its desmethylates between the patients with cancer pain and those with non-cancer pain. The pharmacokinetics may be affected by the pathology, meals, and concomitant drugs. Future clinical studies should assess interindividual variation in tramadol pharmacokinetics in patients treated with oral tramadol. Forth, this method determined the total concentration of tramadol and its desmethylates in human plasma. Although the plasma protein binding of tramadol is approximately 20 % [27], no information on the protein binding of the desmethylates is obtained. Analytical method that determines the free tramadol and its desmethylates would reveal the interindividual variation in tramadol pharmacokinetics.

## Conclusions

A simultaneous and isocratic LC-MS/MS method for the determination of tramadol and its desmethylates in human plasma has been established. This method possesses an acceptable degree of precision and accuracy in accordance with international guidelines [19]. This analytical method can be helpful for evaluating the pharmacokinetics of oral tramadol, including the determination of its desmethylates, in patients with cancer pain or non-cancer pain.

## Abbreviations

CYP: Cytochrome P450
IQR: Interquartile range
IS: Internal standard
LC: Liquid chromatography
LLOQ: Lower limit of quantification
MS/MS: Tandem mass spectrometry
NDT: *N*-desmethyltramadol
NODT: *N,O*-didesmethyltramadol
ODT: *O*-desmethyltramadol
pKa: Acid dissociation constant
QC: Quality control
RSD: Relative standard deviation
SD: Standard deviation

## References

[1] Vickers MD, O’Flaherty D, Szekely SM, Read M, Yoshizumi J (1992). Tramadol: pain relief by an opioid without depression of respiration. Anaesthesia.

[2] Hennies HH, Friderichs E, Schneider J (1988). Receptor binding, analgesic and antitussive potency of tramadol and other selected opioids. Arzneimittelforschung.

[3] Scott LJ, Perry CM (2000). Tramadol, a review of its use in perioperative pain. Drugs.

[4] Beakley BD, Kaye AM, Kaye AD (2015). Tramadol, pharmacology, side effects, and serotonin syndrome: a review. Pain Physician.

[5] Gibson TP (1996). Pharmacokinetics, efficacy, and safety of analgesia with a focus on tramadol HCl. Am J Med.

[6] Lintz W, Barth H, Becker R, Frankus E, Schmidt-Bothelt E (1998). Pharmacokinetics of tramadol and bioavailability of enteral tramadol formulations. 2nd communication: drops with ethanol. Arneimittelforschung.

[7] Paar WD, Poche S, Gerloff J, Dengler HJ (1997). Polymorphic CYP2D6 mediates O-demethylation of the opioid analgesic tramadol. Eur J Clin Pharmacol.

[8] Gillen C, Haurand M, Kobelt DJ, Wnendt S (2000). Affinity, potency and efficacy of tramadol and its metabolites at the cloned human μ-opioid receptor. Naunyn Schmiedebergs Arch Pharmacol.

[9] Gan SH, Ismail R, Wan Adnan WA, Wan Z (2002). Correlation of tramadol pharmacokinetics and CYP2D6*10 genotype in Malaysian subjects. J Pharm Biomed Anal.

[10] Gan SH, Ismail R (2001). Validation of a high-performance liquid chromatography method for tramadol and *O*-desmethyltramadol in human plasma using solid-phase extraction. J Chromatogr B Biomed Sci Appl.

[11] Ardakani YH, Rouini MR (2007). Improved liquid chromatographic method for the simultaneous determination of tramadol and its three main metabolites in human plasma, urine and saliva. J Pharml Biomed Anal.

[12] Patel BN, Sharma N, Sanyal M, Shrivastav PS (2009). An accurate, rapid and sensitive determination of tramadol and its active metabolite *O*-desmethyltramadol in human plasma by LC–MS/MS. J Pharm Biomed Anal.

[13] Giebułtowicz J, Piotrowski R, Baran J, Kułakowski P, Wroczyński P (2016). Application of a novel liquid chromatography/tandem mass spectrometry method for the determination of antazoline in human plasma: Result of ELEPHANT-I [ELEctrophysiological, pharmacokinetic and hemodynamic effects of PHenazolinum (ANTazoline mesylate)] human pharmacokinetic study. J Pharm Biomed Anal.

[14] Georgită C, Sora I, Albu F, Monciu CM (2010). Comparison of a LC/MS method with a LC/UV method for the determination of metformin in plasma samples. Farmacia.

[15] Gan SH, Ismail R, Wan Adnan WA, Wan Z (2002). Method development and validation of a high-performance liquid chromatographic method for tramadol in human plasma using liquid-liquid extraction. J Chromatogr B.

[16] Gu Y, Fawcett JP (2005). Improved HPLC method for the simultaneous determination of tramadol and *O*-desmethyltramadol in human plasma. J Chromatogr B.

[17] Cvan TK, Kerec KM, Trontelj J, Grabnar I, Tschirner A, Palus S, Anker SD, Springer J, Lainscak M (2015). Influence of cancer cachexia on drug liver metabolism and renal elimination in rats. J Cachexia Sarcopenia Muscle.

[18] Fearon K, Arends J, Baracos V (2013). Understanding the mechanisms and treatment options in cancer cachexia. Nat Rev Clin Oncol.

[19] U.S. Food and Drug Administration, Guidance for Industry Bioanalytical Method Validation. (2001) Available from: http://www.fda.gov/downloads/Drugs/GuidanceComplianceRegulatoryInformation/Guidances/ucm070107.pdf#search=’GuidanceforIndustryBioanalyticalMethodValidation. Accessed 18 Aug 2016.

[20] Nobilis M, Kopecký J, Kvetina J, Chládek J, Svoboda Z, Vorísek V, Perlík F, Pour M, Kuneš J (2002). High-performance liquid chromatographic determination of tramadol and its *O*-desmethylated metabolite in blood plasma: application to a bioequivalence study in humans. J Chromatogr A.

[21] Meyer MR, Rosenborg S, Stenberg M, Beck O (2015). First report on the pharmacokinetics of tramadol and O-desmethyltramadol in exhaled breath compared to plasma and oral fluid after a single oral dose. Biochem Pharmacol.

[22] Haage P, Kronstrand R, Carlsson B, Kugelberg FC, Josefsson M (2016). Quantitation of the enantiomers of tramadol and its three main metabolites in human whole blood using LC-MS/MS. J Pharm Biomed Anal.

[23] Lewis KS, Han NH (1997). Tramadol: a new centrally acting analgesic. Am J Health Syst Pharm.

[24] Grond S, Sablotzki A (2004). Clinical pharmacology of tramadol. Clin Pharmacokinet.

[25] Naito T, Tashiro M, Yamamoto K, Ohnishi K, Kagawa Y, Kawakami J (2012). Impact of cachexia on pharmacokinetic disposition of and clinical responses to oxycodone in cancer patients. Eur J Clin Pharmacol.

[26] Naito T, Tashiro M, Ishida T, Ohnishi K, Kawakami J (2013). Cancer cachexia raises the plasma concentration of oxymorphone through the reduction of CYP3A but not CYP2D6 in oxycodone-treated patients. J Clin Pharmacol.

[27] Lee CR, McTavish D, Sorkin EM (1993). Tramadol. A preliminary review of its pharmacodynamic and pharmacokinetic properties, and therapeutic potential in acute and chronic pain states. Drugs.

